# Early Postpartum Milk-Expression and Expressed-Milk Feeding Practices in Relation to Breastfeeding Outcomes: A Systematic Review

**DOI:** 10.3390/healthcare14121755

**Published:** 2026-06-18

**Authors:** Maria Dagla, Panagiota Brani, Calliope Dagla, Eleni Tsolaridou, Sevasti Louverdi, Evangelia Antoniou, Eirini Tomara

**Affiliations:** Laboratory of Midwifery Care During Antenatal and Post Natal Period-Breastfeeding, Department of Midwifery, School of Health & Care Sciences, University of West Attica, 12243 Athens, Greece; brani100@yahoo.gr (P.B.); daglakalliopi@uniwa.gr (C.D.); slouverdi@uniwa.gr (S.L.); lilanton@uniwa.gr (E.A.); etomara@uniwa.gr (E.T.)

**Keywords:** breast milk expression, breast pump use, early postpartum period, breastfeeding outcomes, breastfeeding duration, exclusive breastfeeding, expressed breast milk feeding, lactation, systematic review, maternal–infant feeding practices

## Abstract

**Background/Objectives**: Early postpartum milk expression is common, but its relationship with later breastfeeding outcomes depends on the clinical context in which expression is used. This systematic review examined distinct exposure domains, including expression method, timing and intensity of expression, expressed-milk feeding modality, composite in-hospital feeding patterns, and clinically indicated expression in pump-dependent populations. **Methods**: Following PRISMA 2020 guidance, primary studies of postpartum milk expression, breast pump use, or expressed breast-milk feeding were identified and synthesized using a PECO framework. Because included studies differed substantially in population, exposure definition, comparator structure, and outcome measurement, meta-analysis was not undertaken. Instead, studies were grouped a priori into exposure families, and conclusions were derived within each domain while explicitly considering risk of bias was assessed using RoB 2, version of 22 August 2019, for randomized trials and the original 2016 version of ROBINS-I for non-randomized studies. **Results**: Seventeen studies met the eligibility criteria. In healthy term dyads, routine early pumping or exclusive/predominant expressed-milk feeding was generally associated with less favorable breastfeeding continuation than direct feeding at the breast, although these associations were mainly derived from observational studies at serious risk of confounding by indication. In preterm, separated, or otherwise pump-dependent dyads, earlier and more frequent milk expression was associated with improved early lactation outcomes, including lactogenesis, milk yield, or achievement of full lactation; evidence for longer-term breastfeeding continuation was more limited. Broad population-level definitions of any pump use produced less consistent findings and were not directly comparable with studies of exclusive expressed-milk feeding. **Conclusions**: Early postpartum milk expression should be interpreted as a context-dependent practice rather than as a uniform exposure. The strongest clinical inference is that direct breastfeeding should be supported when feasible in healthy term dyads, whereas early and frequent expression remains necessary and potentially beneficial when direct breastfeeding is not immediately possible.

## 1. Introduction

Breastfeeding and the provision of human milk remain the clinical reference standards for infant feeding and neonatal nutrition [1]. The World Health Organization (WHO) recommends initiation of breastfeeding within the first hour after birth, exclusive breastfeeding for the first 6 months of life, and continued breastfeeding thereafter with appropriate complementary feeding [2,3]. The American Academy of Pediatrics likewise recommends exclusive breastfeeding for approximately 6 months and continuation for 2 years or beyond, as mutually desired by mother and infant [1]. Because lactation establishment occurs during the immediate postpartum period, early maternity care practices are likely to influence subsequent breastfeeding trajectories [4]. Accordingly, the WHO/UNICEF Ten Steps to Successful Breastfeeding emphasize immediate skin-to-skin contact, support for early initiation of breastfeeding, and avoidance of unnecessary supplementation [5,6,7,8]. The Academy of Breastfeeding Medicine similarly states that indications for supplementation in healthy term infants are few and should be based on clinical assessment rather than routine practice [9].

Within this clinical framework, breast milk expression by hand or pump has become a common component of postpartum infant-feeding practice [10]. In U.S. data, breast milk expression was described as a very common practice and was associated strongly with maternal employment [10]. Prospective cohort data from Australia further showed that breast pump use was common among mothers of healthy term infants from birth to 6 months postpartum [11]. These observations indicate that milk expression is not limited to neonatal intensive care settings or medically complex dyads.

To avoid ambiguity, this review uses the following terminology consistently. Milk expression refers to removal of milk from the breast without direct infant suckling and includes both hand expression and pump-based expression. Hand expression refers to milk removal using the mother’s hands. Manual pumping refers to milk removal using a non-electric breast pump. Electric pumping refers to milk removal using an electrically powered breast pump, including hospital-grade pumps when specified. Routine pumping strategies refer to planned or scheduled early postpartum expression rather than expression initiated only in response to a specific clinical indication. Direct breastfeeding refers to feeding at the breast. Expressed-milk feeding refers to feeding previously expressed human milk to the infant by bottle, cup, syringe, or another non-breast route. Exclusive expressed-milk feeding denotes expressed milk as the sole human-milk feeding mode without direct breastfeeding at the breast. Mixed direct-plus-expressed feeding denotes both direct breastfeeding and expressed-milk feeding. These definitions distinguish the method of milk removal from the mode of infant feeding.

The clinical indications for early postpartum milk expression are heterogeneous and include cesarean delivery, maternal–infant separation, ineffective latch, poor milk transfer, delayed secretory activation, prematurity, and maternal intention to store milk [12,13,14,15]. This heterogeneity is methodologically important because the association between milk expression and later breastfeeding outcomes may be affected by confounding by indication [12,16]. In pump-dependent or preterm populations, early and frequent milk removal may be necessary to establish or preserve milk production when direct breastfeeding is not feasible [16]. By contrast, in healthy term dyads, routine early pumping or early substitution of direct breastfeeding with expressed milk feeding may have different implications for later breastfeeding continuation [12,17,18].

Primary studies and prior reviews have not evaluated a single homogeneous intervention. In healthy term dyads, experimental and cohort evidence has compared routine early pumping, hand expression, electric pumping, direct breastfeeding, exclusive expressed-milk feeding, and broader pump use, with findings varying according to comparator and outcome definition [12,13,14,15,16,17,18,19,20]. Studies of exclusive or predominant expressed-milk feeding generally suggest shorter breastfeeding continuation than direct feeding at the breast, whereas broader population-level measures of any pump use have sometimes suggested longer duration, probably reflecting differences in indication, pump access, and underlying maternal–infant circumstances [16,17,18,20].

Evidence from preterm or pump-dependent settings has a different clinical meaning because expression is often needed to establish or preserve lactation when direct breastfeeding is not immediately feasible [21,22,23]. Thus, the gap in the literature is not simply that previous findings are inconsistent; rather, clinically different practices have often been combined under broad labels such as milk expression, breast pumping, or expressed-milk feeding. A systematic review that separates method of milk removal, timing and intensity of expression, feeding modality, and population context is therefore needed before breastfeeding outcomes can be interpreted coherently.

Not all studies, however, support a uniformly adverse interpretation of pump use. Using PRAMS data, Nardella et al. reported that pump use was associated with longer breastfeeding duration overall, although the magnitude of association differed across racial and ethnic groups [16]. This inconsistency likely reflects differences in exposure definition, indication for pumping, comparator selection, and outcome ascertainment across studies [12,16]. In particular, “any pump use” in surveillance data is not methodologically equivalent to routine early postpartum pumping, exclusive expressed milk feeding, or clinically indicated expression in dyads with feeding difficulty or maternal–infant separation.

A major unresolved issue is therefore whether different forms of early postpartum milk expression have distinct relationships with breastfeeding continuation depending on population, indication, and feeding modality, rather than whether milk expression as a broad category is uniformly beneficial or harmful. The evidence base is therefore characterized by substantial clinical and methodological heterogeneity rather than by a simple contrast between milk expression and no expression. Studies differ in population type, exposure window, expression method, comparator structure, and outcome definition, ranging from early lactation parameters to later breastfeeding cessation. These differences are clinically meaningful: in healthy term dyads, milk expression may operate as an alternative to direct feeding at the breast, whereas in preterm, separated, or otherwise pump-dependent dyads it may be the only feasible strategy for establishing or preserving lactation. Consequently, the present review was structured around conceptually distinct early postpartum exposure families: (1) milk-expression methods, such as hand expression, manual pumping, and electric pumping; (2) timing and intensity of expression, including time to first expression and frequency of expression; (3) expressed-milk feeding modalities, including exclusive expressed breast-milk feeding and mixed direct-at-breast plus expressed-milk feeding; and (4) composite in-hospital feeding patterns involving direct breastfeeding, expressed breast milk, and/or formula. Studies in preterm or pump-dependent populations were considered as a clinically distinct lactation-establishment or lactation-preservation context. The review question was therefore addressed through pre-specified subquestions, with breastfeeding outcomes interpreted within each comparison framework rather than across all exposure types as though they were analytically interchangeable.

## 2. Materials and Methods

### 2.1. Study Design and Review Question

This study was conducted as a systematic review of primary studies evaluating the association between early postpartum milk-expression practices, expressed-milk feeding practices, and breastfeeding outcomes. The review was designed and reported in accordance with the Preferred Reporting Items for Systematic Reviews and Meta-Analyses (PRISMA 2020) statement [24].

Because the literature in this field evaluates multiple non-equivalent exposure constructs, the review was designed a priori as a PECO-based review with pre-specified exposure families and corresponding subquestions, rather than as a conventional single-exposure PICO review. The population comprised postpartum mothers and their newborn infants. The exposure domain included early postpartum milk-expression and expressed-milk feeding practices occurring during the first postpartum days, the postpartum hospital stay, or an explicitly defined early postpartum lactation-establishment period, depending on the design of the included study. Comparator groups included direct breastfeeding at the breast only, no milk expression, later or less frequent expression, or alternative feeding or milk-expression patterns, according to the clinical question addressed by each study. Outcomes included breastfeeding duration, time to breastfeeding cessation, duration of exclusive breastfeeding, continuation of any breastfeeding, delayed lactogenesis II, milk volume, and achievement of full lactation. Throughout the manuscript, terminology is aligned with this framework: milk expression is used for milk-removal practices, pump use is reserved for studies that specifically measured pump-based expression, and expressed-milk feeding is used for the infant feeding modality rather than the method of milk removal.

To preserve conceptual clarity, the following exposure families and review subquestions were specified in advance:

Subquestion 1: Expression method in healthy term dyads.

Among healthy term mother–infant dyads, how are different early milk-removal methods (e.g., hand expression, manual pumping, electric pumping, or routine pumping strategies) associated with subsequent breastfeeding outcomes?

Subquestion 2: Timing and intensity of expression.

Among postpartum mothers, particularly in pump-dependent or separated dyads, how are earlier initiation of milk expression and greater expression frequency associated with breastfeeding-related and lactation-related outcomes?

Subquestion 3: Expressed-milk feeding modality in healthy term dyads.

Among healthy term mother–infant dyads, how are exclusive expressed breast milk feeding and mixed direct-at-breast plus expressed-milk feeding associated with breastfeeding duration or cessation compared with direct feeding at the breast?

Subquestion 4: Composite in-hospital feeding patterns.

During the postpartum hospital stay, how are composite feeding patterns involving direct breastfeeding, expressed breast milk, and/or formula associated with later breastfeeding outcomes?

Subquestion 5: Pump-dependent or preterm populations.

In preterm or otherwise pump-dependent populations, how are early and frequent milk-expression practices associated with lactation establishment, milk production, and subsequent breastfeeding-related outcomes?

These subquestions were intended to avoid treating milk-removal methods, feeding modalities, and clinically indicated pump-dependent expression as though they represented a single exposure with a single overall clinical meaning. Accordingly, synthesis and interpretation were conducted within these pre-specified exposure families rather than across all included studies as a single pooled question.

### 2.2. Eligibility Criteria

Studies were eligible if they met all of the following criteria:

(1) Primary research studies;

(2) Randomized controlled trials, non-randomized interventional studies, prospective cohort studies, retrospective cohort studies, or case–control studies;

(3) Postpartum mothers and newborn infants as the study population;

(4) Evaluation of at least one early postpartum milk-expression or expressed-milk feeding practice, including milk-expression method, timing of first expression, frequency or intensity of expression, exclusive or mixed expressed breast milk feeding, or in-hospital feeding patterns involving direct breastfeeding, expressed breast milk, and/or formula, during the first 24–48 h postpartum, the postpartum hospital stay, or the early postpartum period;

(5) Reporting of at least one breastfeeding-related outcome;

(6) Publication as full-text articles in English.

Studies were excluded if they were reviews, scoping reviews, editorials, commentaries, protocols, conference abstracts without full text, case reports, dissertations, or non-English publications. Studies were also excluded if they did not evaluate early postpartum milk expression as a relevant exposure or did not report outcomes pertinent to breastfeeding continuation, breastfeeding duration, or early lactation. Eligibility was determined with reference to the pre-specified exposure families described in Section 2.1. Studies were not assumed to address the same clinical question solely because they involved breast milk expression; rather, each study was classified according to the principal exposure construct evaluated and was interpreted within the corresponding subquestion framework.

### 2.3. Information Sources

A systematic literature search was conducted in the following databases: PubMed/MEDLINE, Scopus, and CINAHL. These databases were selected to capture biomedical, nursing, lactation, and public health literature relevant to maternal–infant feeding. The electronic database search was conducted from database inception to 31 January 2026. The final search was run during December 2025–January 2026. No date restriction was applied at the search stage. Only full-text articles published in English were considered eligible at the screening stage. The final review included 17 studies.

### 2.4. Search Strategy

The search strategy was developed around three concept domains:

(1) Breast milk expression or pumping;

(2) Early postpartum timing;

(3) Breastfeeding outcomes.

Free-text terms and database-specific field tags were used. In PubMed, title/abstract field searching can be performed using [Title/Abstract] or the abbreviated [tiab] field tag. In EBSCO databases such as CINAHL, two-character field codes such as TI for title, AB for abstract, and SU for subject terms are supported. In Scopus, the standard advanced-search field code for simultaneous title, abstract, and keyword searching is TITLE-ABS-KEY.

The search strategy was adapted to the indexing and syntax requirements of each database. Manual screening of the reference lists of relevant articles was additionally performed to identify any eligible studies not retrieved through electronic searching.

### 2.5. Database-Specific Boolean Search Strategies

#### 2.5.1. PubMed/MEDLINE

The PubMed search strategy was constructed using title/abstract field tags and Boolean operators: *(“breast milk expression”[Title/Abstract] OR “milk expression”[Title/Abstract] OR “expressed breast milk”[Title/Abstract] OR “breast pump”[Title/Abstract] OR pumping[Title/Abstract] OR “breast pumping”[Title/Abstract] OR “hand expression”[Title/Abstract]) AND (postpartum[Title/Abstract] OR “post-partum”[Title/Abstract] OR “early postpartum”[Title/Abstract] OR “immediate postpartum”[Title/Abstract] OR “postpartum hospital stay”[Title/Abstract] OR “first 24 h”[Title/Abstract] OR “first 48 h”[Title/Abstract] OR “first 72 h”[Title/Abstract] OR “first days”[Title/Abstract] OR “after delivery”[Title/Abstract]) AND (“breastfeeding duration”[Title/Abstract] OR “breastfeeding outcomes”[Title/Abstract] OR “breastfeeding cessation”[Title/Abstract] OR “exclusive breastfeeding”[Title/Abstract] OR “breastfeeding continuation”[Title/Abstract] OR lactation[Title/Abstract] OR “lactogenesis II”[Title/Abstract] OR “milk volume”[Title/Abstract])*.

A functionally equivalent version using the abbreviated PubMed field tag was also acceptable: *(“breast milk expression”[tiab] OR “milk expression”[tiab] OR “expressed breast milk”[tiab] OR “breast pump”[tiab] OR pumping[tiab] OR “breast pumping”[tiab] OR “hand expression”[tiab]) AND (postpartum[tiab] OR “early postpartum”[tiab] OR “immediate postpartum”[tiab] OR “postpartum hospital stay”[tiab] OR “first 24 h”[tiab] OR “first 48 h”[tiab] OR “first 72 h”[tiab] OR “first days”[tiab] OR “after delivery”[tiab]) AND (“breastfeeding duration”[tiab] OR “breastfeeding outcomes”[tiab] OR “breastfeeding cessation”[tiab] OR “exclusive breastfeeding”[tiab] OR “breastfeeding continuation”[tiab] OR lactation[tiab] OR “lactogenesis II”[tiab] OR “milk volume”[tiab]).*

#### 2.5.2. Scopus

The Scopus search strategy used the TITLE-ABS-KEY field code: *TITLE-ABS-KEY(“breast milk expression” OR “milk expression” OR “expressed breast milk” OR “breast pump” OR pumping OR “breast pumping” OR “hand expression”) AND TITLE-ABS-KEY(postpartum OR “early postpartum” OR “immediate postpartum” OR “postpartum hospital stay” OR “first 24 h” OR “first 48 h” OR “first 72 h” OR “first days” OR “after delivery”) AND TITLE-ABS-KEY(“breastfeeding duration” OR “breastfeeding outcomes” OR “breastfeeding cessation” OR “exclusive breastfeeding” OR “breastfeeding continuation” OR lactation OR “lactogenesis II” OR “milk volume”).*

#### 2.5.3. CINAHL

The CINAHL search strategy was adapted to EBSCO field-code syntax. A title/abstract-focused search could be written as follows: *(TI “breast milk expression” OR AB “breast milk expression” OR TI “milk expression” OR AB “milk expression” OR TI “expressed breast milk” OR AB “expressed breast milk” OR TI “breast pump” OR AB “breast pump” OR TI pumping OR AB pumping OR TI “breast pumping” OR AB “breast pumping” OR TI “hand expression” OR AB “hand expression”) AND (TI postpartum OR AB postpartum OR TI “early postpartum” OR AB “early postpartum” OR TI “immediate postpartum” OR AB “immediate postpartum” OR TI “postpartum hospital stay” OR AB “postpartum hospital stay” OR TI “first 24 h” OR AB “first 24 h” OR TI “first 48 h” OR AB “first 48 h” OR TI “first 72 h” OR AB “first 72 h” OR TI “first days” OR AB “first days” OR TI “after delivery” OR AB “after delivery”) AND (TI “breastfeeding duration” OR AB “breastfeeding duration” OR TI “breastfeeding outcomes” OR AB “breastfeeding outcomes” OR TI “breastfeeding cessation” OR AB “breastfeeding cessation” OR TI “exclusive breastfeeding” OR AB “exclusive breastfeeding” OR TI “breastfeeding continuation” OR AB “breastfeeding continuation” OR TI lactation OR AB lactation OR TI “lactogenesis II” OR AB “lactogenesis II” OR TI “milk volume” OR AB “milk volume”).*

If a broader subject-heading-supported strategy was required in CINAHL, subject-term searching using SU could be combined with title and abstract searching, depending on local database configuration.

### 2.6. Study Selection

Study selection was conducted in two stages: title/abstract screening followed by full-text eligibility assessment. Two reviewers independently screened all records at both stages against the predefined eligibility criteria. Disagreements were resolved through discussion and consensus; when consensus could not be reached, a third reviewer adjudicated. Reasons for exclusion at the full-text stage were recorded. Reference-management software was used only to detect and remove duplicate records. No automation tool, machine-learning classifier, or automated eligibility rule was used to exclude records; all screening exclusions were made by reviewers through application of the eligibility criteria. The selection process was documented in a PRISMA 2020 flow diagram.

### 2.7. Data Extraction

Data extraction was performed using a standardized, pilot-tested data-extraction form. Two reviewers independently extracted data from each included study. Extracted items included study identification, setting, design, sample size, population characteristics, gestational category, exposure definition, timing of expression, comparator group, outcome definitions, follow-up duration, principal findings, and major methodological limitations. Any discrepancies in extracted data were resolved through discussion and consensus, with arbitration by a third reviewer if required.

Additional variables considered clinically relevant for interpretation were also extracted, including whether the population consisted of healthy term dyads or preterm/pump-dependent dyads, whether milk expression was clinically indicated, whether direct breastfeeding occurred concurrently, whether expressed milk feeding was exclusive or mixed, and whether formula supplementation was reported.

In addition to standard study characteristics, each included study was classified according to the primary exposure construct evaluated: (1) expression method, (2) timing or frequency of expression, (3) expressed-milk feeding modality, or (4) composite in-hospital feeding pattern. Studies conducted in preterm or otherwise pump-dependent populations were additionally identified as representing lactation-establishment or lactation-preservation contexts. This classification was used to avoid treating conceptually distinct exposure constructs as analytically interchangeable. This classification was pre-specified in the analytic framework and was used to ensure that conceptually distinct exposure constructs were synthesized within their respective review subquestions rather than treated as analytically interchangeable.

### 2.8. Outcomes

The primary outcome was breastfeeding duration, including duration of any breastfeeding, duration of exclusive breastfeeding, or time to breastfeeding cessation, according to the definitions used in the included studies.

The secondary outcomes were breastfeeding continuation at specified follow-up time points, exclusive breastfeeding status, expressed milk feeding patterns, delayed lactogenesis II, milk volume, successful full lactation, and other early lactation-related outcomes.

### 2.9. Risk-of-Bias Assessment

Risk of bias was assessed at the study level using established design-specific appraisal tools. For randomized controlled trials, risk of bias was evaluated using the Cochrane Risk of Bias 2 (RoB 2) tool. For non-randomized studies of interventions or exposure-based comparisons, risk of bias was assessed using RoB 2, version of 22 August 2019, for randomized trials and the original 2016 version of ROBINS-I for non-randomized studies, with particular attention to bias due to confounding, participant selection, exposure classification, deviations from intended exposures, missing data, outcome measurement, and selective reporting.

Two reviewers independently assessed each included study. Disagreements were resolved through discussion and consensus, with adjudication by a third reviewer if required.

Because the review included clinically and methodologically heterogeneous designs, risk-of-bias judgments were interpreted within study design categories rather than converted into a numerical score or percentage-based summary. No overall quantitative weighting scheme was applied. Instead, risk-of-bias assessments were used to inform the interpretation of findings, with particular attention to confounding by indication, exposure misclassification, comparator appropriateness, and completeness of outcome ascertainment in observational studies.

For studies that were primarily descriptive and did not address a comparative exposure–outcome question, formal risk-of-bias assessment was interpreted cautiously, and such studies were used primarily for contextual characterization of the exposure environment rather than for causal inference regarding breastfeeding outcomes.

### 2.10. Data Synthesis

Quantitative meta-analysis was not performed because the included studies differed substantially in design, population type, gestational category, indication for expression, exposure timing, milk-expression modality, comparator definition, and breastfeeding outcome measurement. Under these conditions, structured narrative synthesis was considered more appropriate than statistical pooling because pooling would have combined non-equivalent exposure constructs and clinically distinct populations.

The synthesis followed a pre-specified three-step decision process. First, each study was assigned to the exposure family and clinical context to which its principal comparison most closely corresponded: expression method, timing or intensity of expression, expressed-milk feeding modality, composite in-hospital feeding pattern, or pump-dependent/preterm lactation establishment. Second, findings were compared only within the relevant exposure family, with attention to population, indication for expression, comparator structure, concurrent direct breastfeeding, formula supplementation, and outcome timing. Third, a domain-level interpretation was derived by considering the direction and consistency of findings, proximity of the outcome to the exposure, biological and clinical plausibility, and the risk-of-bias judgment for the contributing studies.

Risk of bias was not treated as a separate descriptive appendix but was used to qualify the strength of each domain-level conclusion. Findings from randomized trials were interpreted as stronger evidence for short-term intervention effects when the comparator was clinically aligned, whereas observational studies at serious risk of confounding by indication were interpreted as evidence of patterned association rather than definitive causation. Descriptive exposure-characterization studies were used to contextualize how common milk expression was but were not used as causal evidence for breastfeeding duration.

## 3. Results

### 3.1. Study Selection

**Figure 1:**
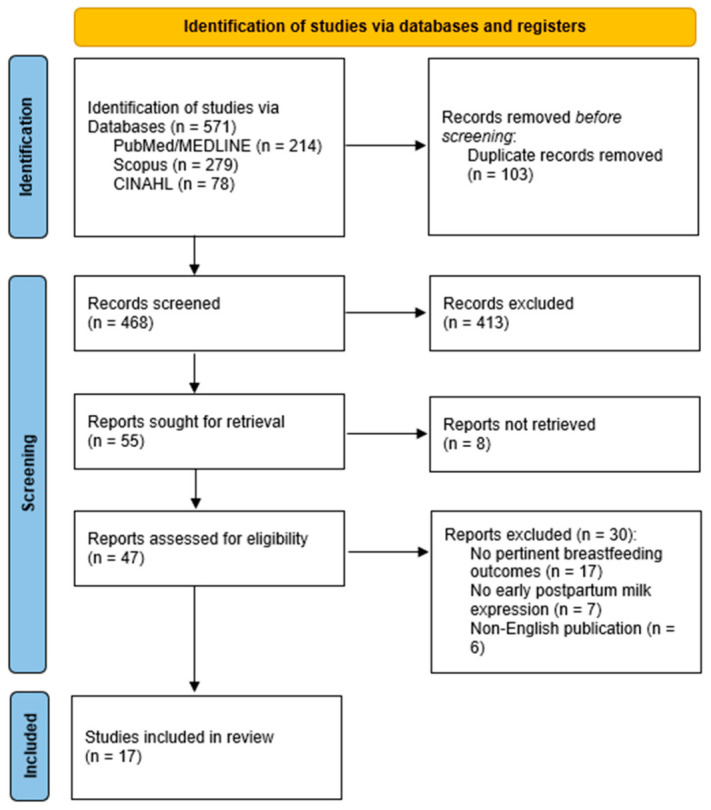
PRISMA flowchart.

The database search identified 571 records before duplicate removal: PubMed/MEDLINE (n = 214), Scopus (n = 279), and CINAHL (n = 78). After removal of 103 duplicate records and exclusion of 339 records marked as ineligible, 129 records remained for title and abstract screening. Following title and abstract screening, 74 records were excluded and 55 reports were sought for retrieval. Eight reports were not retrieved. A total of 47 full-text reports were assessed for eligibility. Of these, 30 reports were excluded because they did not report breastfeeding outcomes pertinent to the review question (n = 17), did not evaluate early postpartum milk expression (n = 7), or were non-English publications (n = 6). Seventeen studies met the eligibility criteria and were included in the final synthesis (Figure 1).

### 3.2. Study Characteristics

Before presentation of the findings, the overall evidence base was summarized by study design, country or setting, population, sample size, and principal exposure construct (Table 1). The 17 included studies comprised randomized clinical trials, comparative clinical studies, prospective cohort studies, longitudinal survey cohorts, population-based observational analyses, and descriptive exposure-characterization studies. They were conducted in the United States, Australia, Japan, China/Hong Kong, Singapore, and the United Kingdom. The populations were grouped analytically into healthy term dyads and preterm, separated, at-risk, or otherwise pump-dependent dyads. Sample sizes ranged from small clinical studies to large population-based cohorts, reinforcing the need to interpret findings within exposure family and clinical context rather than as a single pooled question.

The 17 included studies comprised a heterogeneous evidence base including randomized clinical trials, prospective cohort studies, and other observational designs. Most studies were conducted in healthy term mother–infant dyads, whereas a smaller subgroup focused on preterm or pump-dependent populations in whom direct breastfeeding at the breast was limited or not immediately feasible. The included literature also varied substantially in the way exposure was defined, including any milk expression, breast pump use, exclusive expressed milk feeding, hand expression versus pumping, early in-hospital feeding patterns, and structured early-expression protocols. Outcome definitions were similarly heterogeneous and included breastfeeding duration, time to breastfeeding cessation, breastfeeding status at 2 or 6 months, exclusive breastfeeding duration, lactogenesis II, milk yield, and achievement of full lactation [12,14,15,17,18,19,21,22,23].

Three studies were primarily descriptive and were retained because they characterized the early postpartum exposure environment relevant to the review question. Labiner-Wolfe et al. showed that breast milk expression was a very common practice and was strongly associated with maternal employment [10]. Johns et al. reported that, among healthy term infants assessed at 24–48 h postpartum, 47% had been fully breastfeeding at the breast from birth and another 47% had received at least some expressed breast milk [25]. In a later cohort report, Johns et al. showed that breast pump use among mothers of healthy term infants was common between birth and 6 months postpartum [26]. These studies did not provide the clearest duration-based effect estimates, but they documented that early milk expression and pump availability were common in otherwise low-risk maternity populations.

The included studies did not evaluate a single uniform exposure. Instead, they examined several conceptually distinct constructs, including milk-expression methods, timing and frequency of milk expression, expressed-milk feeding modalities, composite in-hospital feeding patterns, and clinically indicated expression in preterm or pump-dependent settings. These constructs were not treated as analytically interchangeable in the narrative synthesis (Table 2).

### 3.3. Findings in Healthy Term Populations

Among healthy term dyads, early interventional and comparative studies did not support a uniform benefit of routine breast pumping in the immediate postpartum period. In women after cesarean delivery, Chapman et al. found that breast pumping during the first 72 h postpartum did not improve milk transfer during the first 72 h and may negatively affect breastfeeding duration among primiparous women [15]. In mothers of healthy term infants aged 12–36 h with poor feeding, Flaherman et al. reported that hand expression in the early postpartum period was associated with higher breastfeeding rates at 2 months than breast pumping [14]. Ohyama et al. reported that, in the first 48 h after delivery, hospital-grade electric pumping was more effective than manual expression for maximizing available milk volume and was perceived as more comfortable, although that study primarily addressed short-term milk-removal performance rather than later breastfeeding continuation [13].

**Table 1:** Table of Study Characteristics.

Study	Country/Setting	Study Design	Population/Sample Size	Exposure/Timing	Comparator	Main Outcomes	Principal Finding
[15]	United States	Randomized clinical trial	Mothers after cesarean delivery (n = 60)	Breast pumping during the first 24–72 h postpartum	Standard care/no routine pumping	Milk transfer in first 72 h; breastfeeding duration	Breast pumping did not improve milk transfer during the first 72 h and may adversely affect breastfeeding duration among primiparous women.
[27]	Australia	Cohort study	Postpartum mothers from maternity hospitals (n = 587)	Any breast milk expression during the postpartum period	Never expressed milk	Any breastfeeding duration to 6 months	Mothers who expressed milk were less likely to discontinue any breastfeeding before 6 months than those who never expressed milk.
[10]	United States	Observational study	Breastfeeding mothers in a national infant-feeding cohort (IFPS II; n = 4606 overall; n = 1493 with complete 1.5–4.5-month data)	Any, occasional, or regular breast milk expression	None/lower-frequency expression	Prevalence and correlates of expression	Breast milk expression was very common and was associated most strongly with maternal employment.
[13]	Japan	Comparative clinical study	Early postpartum mothers in a tertiary perinatal center (n = 11)	Manual expression versus electric pumping in the first 48 h after delivery	Alternative milk-removal method	Available milk volume; maternal comfort	Electric pumping was more effective for maximizing available milk volume and was reported as more comfortable, although the study primarily addressed short-term lactation performance.
[14]	United States	Randomized trial	Mothers of healthy term infants aged 12–36 h feeding poorly (n = 68)	Hand expression in the early postpartum period	Breast pumping	Breastfeeding at 2 months; expressed milk volume	Hand expression was associated with higher breastfeeding rates at 2 months than breast pumping.
[26]	Australia	Prospective observational study	Healthy term infants and postpartum mothers at 24–48 h (n = 1003)	In-hospital infant feeding practices, including direct breastfeeding and expressed breast milk	Not a formal comparator study	Early feeding practices; prevalence of expressed breast milk exposure	Forty-seven percent of infants had been fully breastfeeding at the breast from birth, and another 47% had received at least some expressed breast milk within 24–48 h.
[12]	China	Prospective cohort study	Mothers of healthy term infants (n = 401 enrolled; n = 345 breastfeeding at 6-week analysis)	Breast milk expression in the early postpartum period; exclusive expressing patterns	Direct breastfeeding/other feeding patterns	Breastfeeding duration	Exclusive expressing in the early postpartum period was associated with shorter breastfeeding duration.
[19]	Australia	Prospective cohort study	Healthy term infants whose mothers intended to breastfeed (n = 1003 recruited; n = 924 followed at 6 months)	Feeding directly at the breast during the postpartum hospital stay (24–48 h)	Any expressed breast milk and/or formula in hospital	Any breastfeeding at 6 months	Infants fed only directly at the breast during the maternity stay were more likely to be breastfeeding at 6 months.
[28]	United States	Longitudinal survey cohort	Mothers pumping human milk postpartum (n = 1116)	Pumping in the early postpartum period; pumping frequency and reasons for pumping	Lower-frequency or different-pattern pumping	Feeding at the breast; exclusively feeding human milk; human milk feeding duration	Higher pumping frequency and nonelective reasons for pumping were associated with shorter durations of feeding at the breast and exclusive human milk feeding.
[25]	Australia	Prospective cohort study	Mothers of healthy term infants (n = 1003)	Breast pump use between birth and 6 months postpartum	Descriptive exposure study	Patterns of pump use	Breast pump use was common among mothers of healthy term infants from birth through 6 months postpartum.
[18]	Hong Kong, China	Prospective cohort study	Healthy full-term infants and mothers (n = 2450 mother–infant pairs)	Exclusive expressed breast-milk feeding during the first 6 months	Direct feeding at the breast	Breast-milk feeding cessation	Exclusive expressed breast-milk feeding was associated with an increased risk of early breast-milk feeding cessation compared with direct feeding at the breast.
[17]	Singapore	Prospective cohort study	Breastfeeding mothers and infants from the GUSTO cohort (n = 541 mother–infant pairs)	Exclusive expressed breast milk feeding or mixed feeding	Direct feeding at the breast	Duration of breastfeeding/early weaning	Mothers who fed expressed breast milk exclusively, but not those practicing mixed feeding, were at higher risk of terminating breastfeeding earlier than mothers who fed directly at the breast.
[21]	Singapore	Randomized trial	At-risk postpartum mothers (n = 60)	Early initiation and regular breast milk expression	Standard care/later or less structured expression	Delayed lactogenesis II	Early initiation and regular breast milk expression reduced the risk of lactogenesis II delay.
[22]	China	Prospective observational study	Mothers of preterm infants using hospital-grade pumps (n = 30)	Exclusive pumping; pumping frequency in early postpartum/NICU context	Lower pumping frequency	Full lactation achievement; milk production	Most mothers pumping at least six times daily with a hospital-grade pump achieved full lactation successfully.
[20]	Hong Kong, China	Cohort study	Mothers of healthy infants (n = 821)	Expressed breast milk feeding during the first 6 months postpartum	Direct breastfeeding/alternative feeding patterns	Breastfeeding duration/cessation	Giving only expressed breast milk was associated with earlier breastfeeding cessation, especially when combined with formula supplementation.
[16]	United States	Population-based observational study (PRAMS)	U.S. lactating population (n = 19,719 mothers; population-weighted cohort)	Any pump use	No pump use	Breastfeeding duration	Pump use was associated with longer breastfeeding duration overall, with variation across racial and ethnic subgroups.
[23]	United Kingdom	Cohort study	Mothers of very preterm infants (n = 132)	First expression of colostrum within 6 h of birth	Later first expression	Milk yield in the first 3 weeks after birth	Mothers who first expressed within 6 h had higher milk yield and greater yield per session during the first 3 weeks; no additional benefit was shown for expression within the first 2 h.

**Table 2:** Mapping of the 17 included studies to the five pre-specified review subquestions.

Study	Population/Context	S1	S2	S3	S4	S5
[15]	Term/cesarean	X				
[27]	Postpartum maternity cohort			X		
[10]	Population-based/contextual		C	C	C	
[13]	Early postpartum mothers	X				
[14]	Healthy term, poor feeding	X				
[26]	Healthy term dyads				X	
[12]	Healthy term dyads			X		
[19]	Healthy term dyads				X	
[28]	Postpartum pumping cohort		X	X		
[25]	Healthy term dyads	C		C		
[18]	Healthy full-term dyads			X		
[17]	Breastfeeding mothers/infants			X		
[21]	At-risk/pump-dependent		X			X
[22]	Preterm/pump-dependent		X			X
[20]	Healthy infant cohort			X		
[16]	Population-based/contextual		C	C		
[23]	Very preterm/pump-dependent		X			X

Note. S1 = expression method in healthy term dyads; S2 = timing and intensity of expression; S3 = expressed-milk feeding modality in healthy term dyads; S4 = composite in-hospital feeding patterns; S5 = pump-dependent or preterm populations. X indicates a primary contribution to the subquestion; C indicates contextual or cross-cutting evidence.

Studies evaluating subsequent breastfeeding duration or continuation in term populations yielded mixed but clinically interpretable findings. Win et al. reported that mothers who expressed milk were less likely to discontinue any breastfeeding before 6 months than mothers who had never expressed milk [27]. In contrast, Jiang et al. reported that exclusive expressing in the early postpartum period was associated with shorter breastfeeding duration [12]. Forster et al. found that healthy term infants fed only directly at the breast 24–48 h after birth were more likely to be continuing to breastfeed at 6 months than infants who received any expressed breast milk and/or formula during the early postpartum period [19]. Taken together, these studies suggest that the effect of milk expression in healthy term populations depends substantially on how the exposure is defined; “any expression” was not equivalent to “exclusive expressing” or to in-hospital replacement of direct breastfeeding with expressed-milk feeding.

Subsequent cohort studies reinforced the distinction between direct breastfeeding at the breast and exclusive or predominant expressed-milk feeding. Bai et al. found that exclusive expressed-milk feeding in healthy full-term infants was associated with an increased risk of early breast-milk feeding cessation compared with direct feeding at the breast [18]. Pang et al. reported that mothers who fed expressed breast milk exclusively, but not those practicing mixed feeding, were at higher risk of terminating breastfeeding earlier than mothers who fed directly at the breast [17]. Fan et al. reported that, in the first 6 months postpartum, giving only expressed breast milk was associated with early breastfeeding cessation, particularly among participants who also supplemented with infant formula [20]. These studies were directionally consistent in indicating that exclusive or predominant expressed-milk feeding was associated with less favorable breastfeeding continuation than direct feeding at the breast.

Felice et al. extended this literature by showing that, among mothers who pumped between 1.5 and 4.5 months postpartum, higher pumping frequency and nonelective reasons for pumping were associated with shorter durations of any human milk feeding, exclusive human milk feeding, and feeding at the breast [28]. This finding is relevant to interpretation of the early postpartum literature because it suggests that pumping intensity and the underlying indication for pumping may be important determinants of subsequent breastfeeding trajectories.

Not all included studies supported an adverse association between pump use and breastfeeding duration. In PRAMS data, Nardella et al. reported that pump use was associated with longer breastfeeding duration overall, with the greatest magnitudes of association observed among non-Hispanic Black and Native American participants [16]. This finding was different from the studies of exclusive expressed-milk feeding and from the in-hospital direct-versus-expressed feeding comparisons, again suggesting that broad population-level “pump use” is not methodologically equivalent to early routine pumping or exclusive expressed-milk feeding.

### 3.4. Findings in Preterm or Pump-Dependent Populations

The three preterm or pump-dependent studies were clinically distinct from the healthy term studies because milk expression functioned primarily as a lactation-establishment or lactation-preservation strategy rather than as an alternative to direct breastfeeding in an otherwise healthy dyad. Fok et al. reported in at-risk Singaporean mothers that early initiation and regular breast milk expression reduced the risk of delayed lactogenesis II [21]. Ru et al. reported in mothers of preterm infants using hospital-grade pumps that most mothers pumping at least six times per day could achieve full lactation successfully [22]. Levene et al. reported that mothers of very preterm infants who first expressed within 6 h of birth had higher milk yield and greater yield per expressing session during the first 3 weeks after birth, without evidence of additional benefit from expression within the first 2 h [23]. These studies were consistent in indicating benefit of early and frequent milk expression in populations in which direct feeding at the breast was limited or not immediately possible.

### 3.5. Overall Pattern of Evidence

Across the included studies, the direction of association depended on the exposure construct and population context. In healthy term dyads, studies of expression method did not demonstrate a consistent advantage of routine early pumping over direct breastfeeding or hand expression. Studies of feeding modality were more consistent: exclusive or predominant expressed breast-milk feeding was generally associated with shorter breastfeeding duration or earlier cessation than direct feeding at the breast. Composite in-hospital feeding studies similarly suggested more favorable longer-term outcomes when infants were fed directly at the breast during the postpartum stay rather than receiving expressed breast milk and/or formula.

**Figure 2:**
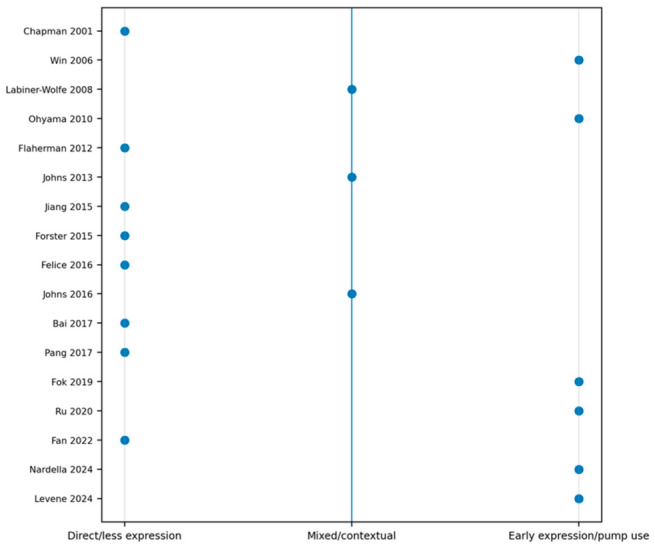
Direction-of-effect plot of the 17 included studies. Studies were categorized according to whether their principal findings favored direct breastfeeding/less expression, favored early expression or pump use, or were mixed/contextual. Classification was based on the principal direction of association and interpreted within the exposure-family framework [10,12,13,14,15,16,17,18,19,20,21,22,23,25,26,27,28].

This pattern did not extend to preterm or otherwise pump-dependent dyads. In these populations, expression was usually a lactation-establishment or lactation-preservation strategy rather than an alternative to effective direct breastfeeding. Earlier initiation and greater frequency of expression were therefore associated with improved early lactation outcomes. Broad population-level studies defining exposure as any or ever pump use yielded less consistent findings, probably because pump use may indicate either clinical need, access to lactation resources, maternal employment planning, or a strategy that supports continued human-milk provision under difficult circumstances (Figure 2) [12,16,17,18,19,20,21,22,23].

### 3.6. Structured Evidence Integration Across Exposure Families

The structured evidence integration is presented at the exposure-family level in Table 3. This table summarizes the principal findings, population context, and risk-of-bias implications within each domain, while reserving broader explanatory interpretation and clinical implications for the Discussion. At the results level, the observed direction of association varied by clinical purpose and population context. When expression functioned mainly as a replacement for direct breastfeeding in healthy term dyads, the included studies generally showed less favorable breastfeeding-continuation outcomes. When expression functioned as lactation establishment or preservation in preterm or pump-dependent dyads, earlier and more frequent expression was associated with more favorable early lactation outcomes. Because most non-randomized comparative evidence remained at serious risk of bias, these findings are reported as patterned associations rather than causal estimates.

### 3.7. Risk-of-Bias Findings

Risk-of-bias judgments directly informed the strength of interpretation. The randomized trials were generally judged as having some concerns, mainly because of limitations related to deviations from intended intervention, incomplete intervention detail, or uncertainty regarding selective reporting. The non-randomized comparative studies were predominantly judged to be at serious risk of bias, principally because exposure to pumping or expressed-milk feeding was strongly linked to clinical circumstances such as cesarean birth, infant feeding difficulty, maternal–infant separation, prematurity, or anticipated return to work. Consequently, findings from healthy term observational cohorts were interpreted as consistent associations rather than as definitive causal effects. Descriptive studies were used only to characterize the exposure environment, whereas preterm and pump-dependent studies were interpreted primarily in relation to early lactation establishment rather than long-term breastfeeding continuation (Table 4).

## 4. Discussion

The principal interpretive issue in this review is that the included studies did not address a single homogeneous exposure. For that reason, this review was designed and synthesized using pre-specified exposure families and corresponding subquestions rather than treating all forms of breast milk expression as analytically equivalent. This framework was important because milk-removal methods, expressed-milk feeding modalities, composite in-hospital feeding patterns, and clinically indicated expression in pump-dependent settings differ in biological meaning, behavioral implications, and susceptibility to confounding. When the evidence is interpreted within these predefined comparison domains, much of the apparent inconsistency across studies becomes clinically and methodologically understandable. In healthy term mother–infant dyads, the most consistent pattern was that direct breastfeeding at the breast was associated with more favorable breastfeeding continuation outcomes than exclusive or predominant expressed milk feeding. By contrast, in preterm or pump-dependent populations, earlier and more frequent expression was generally associated with more favorable early lactation outcomes. This divergence indicates that “breast milk expression” is not a single exposure with a single clinical meaning, but rather a group of distinct practices that operate differently in different maternal–infant settings [11,16,29].

Viewed across domains, the evidence resolves into three clinically distinct patterns. First, in healthy term dyads, early replacement of direct breastfeeding with exclusive or predominant expressed-milk feeding was repeatedly associated with less favorable breastfeeding continuation, although causal certainty was limited by confounding. Second, in preterm, separated, or pump-dependent dyads, expression was not a competing feeding mode but a necessary mechanism of milk removal; in that setting, earlier and more frequent expression was associated with better early lactation outcomes. Third, broad population-level pump-use measures were too heterogeneous to support a single clinical conclusion and were interpreted as indicators of access, need, or adaptive feeding strategy rather than as a uniform intervention.

The term-infant studies in the present review were found to be more consistent than they may initially appear once exposure definitions were examined closely. Chapman et al. did not show a benefit of routine early pumping after cesarean delivery on early milk transfer and raised concern about possible adverse effects on breastfeeding duration among primiparous women [15]. Flaherman et al. found that, in mothers of healthy term infants with early feeding difficulty, hand expression was associated with higher breastfeeding rates at 2 months than breast pumping [14]. Forster et al. showed that infants fed directly at the breast during the postpartum hospital stay were more likely to continue breastfeeding at 6 months than infants exposed to expressed breast milk and/or formula during the first 24–48 h [19]. Jiang et al. extended this pattern by showing that exclusive expressing in the early postpartum period was associated with shorter breastfeeding duration [12]. Considered together, these studies do not support a generalized conclusion that early routine pumping improves breastfeeding outcomes in healthy term dyads. Rather, they suggest that replacing direct breastfeeding at the breast with expressed-milk feeding early in the postpartum course may be associated with less favorable longer-term outcomes.

The later observational studies included in this review further reinforce that distinction. Bai et al. linked exclusive expressed-milk feeding with an increased risk of early breast-milk feeding cessation relative to direct feeding [18]. Pang et al. found that exclusive expressed-milk feeding, but not mixed feeding, was associated with earlier termination of breastfeeding compared with direct feeding at the breast [17]. Fan et al. reported that only expressed-milk feeding was associated with earlier breastfeeding cessation, particularly when combined with formula supplementation [20]. These studies are clinically important because they move beyond the broad exposure of “any pump use” and instead examine feeding mode with greater specificity. Their findings suggest that expressed-milk feeding should not be assumed to be functionally equivalent to direct breastfeeding when the outcome of interest is breastfeeding continuation. A plausible interpretation is that early and sustained reliance on expressed milk may alter dyadic feeding behavior, reduce direct breast stimulation by the infant, or mark unresolved feeding difficulty, all of which could influence later breastfeeding trajectories. However, these same studies remain vulnerable to confounding by indication because mothers who express early or exclusively may differ systematically from mothers who feed directly at the breast in ways that are incompletely captured by routine covariate adjustment.

This issue of confounding by indication is central to interpretation of the whole field and probably explains much of the apparent inconsistency across studies. Nardella et al. reported that pump use, defined broadly at the population level, was associated with a 37% lower hazard of breastfeeding cessation and approximately 21 additional weeks of breastfeeding on average [16]. At face value, that finding seems discordant with the findings reported by Jiang et al., Bai et al., Pang et al., and Fan et al. [12,17,18,20]. However, the underlying exposure in the Nardella analysis was “ever pump use” in a large PRAMS-derived population, not exclusive expressed-milk feeding, not routine early pumping in otherwise healthy dyads, and not in-hospital substitution of direct breastfeeding with expressed milk [16]. In addition, pump use in that analysis was more common among mothers with cesarean delivery, low-birth-weight infants, and longer infant hospitalization, suggesting that pump use may simultaneously function as a marker of clinical need and as a resource that supports breastfeeding continuation under challenging circumstances [16]. The contrast between Nardella et al. and the term-dyad studies therefore argues against treating all forms of milk expression as a single exposure category.

The preterm and pump-dependent studies in the present review support this contextual interpretation. Fok et al. found that early initiation and regular breast milk expression reduced the risk of delayed lactogenesis II in at-risk mothers [21]. Ru et al. reported that frequent use of a hospital-grade pump was associated with successful achievement of full lactation among mothers of preterm infants [22]. Levene et al. showed that first expression within 6 h of birth in mothers of very preterm infants was associated with greater milk yield in the first 3 weeks after delivery [23]. These findings should not be collapsed analytically with the healthy term studies as though they were examining the same clinical question. In preterm or separated dyads, expression is often not a substitute for direct breastfeeding but the only feasible means of establishing or preserving milk production. In that setting, early and frequent milk removal is physiologically and clinically necessary, and the favorable direction of effect on lactation outcomes is biologically coherent.

Our findings are broadly consistent with the earlier systematic review by Johns et al. published in 2013 [11]. That review concluded that evidence on the prevalence and outcomes of expressing breast milk among mothers of healthy term infants was limited, that only a small number of papers specifically addressed well term infants, and that findings were contradictory and complicated by imprecise definitions of breastfeeding and breast milk feeding [11]. The present review reaches a similar methodological conclusion but extends it in two ways. First, more recent cohort studies now show a more coherent pattern when exposure is specified as exclusive expressed-milk feeding rather than any expression. Second, newer population-based analyses indicate that broad pump use may still be associated with longer breastfeeding duration overall, especially in settings with high pump availability. Our review therefore both confirms the core uncertainty identified by Johns et al. and refines it by showing that a major source of inconsistency is exposure heterogeneity rather than simple disagreement between studies.

The present review is also concordant, in direction, with the more recent systematic review and meta-analysis by Fan et al. [29]. That review found that any expressed human milk feeding within 3 months postpartum was associated with increased risk of breastfeeding cessation and that only expressed human milk feeding within 3 months and at or beyond 3 months postpartum was associated with still higher risk of cessation [29]. Fan et al. also reported that only seven of thirty-one included studies were rated as good quality, underscoring the continued methodological fragility of the evidence base [29]. Our synthesis aligns with that overall conclusion for healthy term populations: where expressed-milk feeding becomes the predominant feeding mode, subsequent breastfeeding duration appears less favorable than with direct breastfeeding at the breast. The main difference is that our review deliberately retained preterm and pump-dependent studies as a clinically distinct subgroup, which allowed a more explicit demonstration that the direction of association changes when expression is being used to establish lactation in the setting of maternal–infant separation or prematurity.

A further strength of the present synthesis is that it avoids overinterpreting questions that the available literature was not designed to answer. Gandino et al. examined varying mother’s own milk-expression practices and treatments in relation to infant health and growth outcomes and concluded that no expression method or pump type showed clear benefits for breastfeeding rates or infant growth, while evidence on most clinical outcomes remained very limited [30]. Although that review addressed a different clinical question from the present review, it is consistent with our conclusion that evidence about expression practices is highly fragmented and that technical aspects of milk expression should not be assumed to translate directly into improved long-term breastfeeding outcomes [30]. In other words, a method that improves early milk-removal efficiency, comfort, or milk handling does not necessarily improve breastfeeding continuation in healthy term dyads.

Several methodological limitations of the included studies warrant emphasis. First, exposure definitions were highly inconsistent. Some studies classified any pump use as exposure, others evaluated exclusive expressed milk feeding, and others focused on in-hospital feeding mode or on formal pumping interventions. These are not equivalent constructs. Second, comparator groups were often heterogeneous and sometimes combined direct breastfeeding, expressed milk feeding, and formula supplementation in ways that complicate causal interpretation. Third, confounding by indication was pervasive, particularly in observational studies, because mothers who expressed were often those experiencing cesarean delivery, latch problems, low milk transfer, infant prematurity, return-to-work planning, or other factors independently associated with breastfeeding outcomes. Fourth, outcome definitions varied substantially across studies, including any breastfeeding duration, exclusive breastfeeding duration, status at fixed follow-up points, lactogenesis II, milk yield, and full lactation. Fifth, the timing of exposure ascertainment ranged from the first hours after birth to several months postpartum, which limits the extent to which studies can be considered to address the same review question. These limitations support the decision not to pool effect estimates quantitatively in the present review.

The risk-of-bias assessment further emphasizes that most non-randomized comparative studies in this field are best interpreted as being at serious risk of bias, chiefly because exposure to pumping or expressed-milk feeding is rarely allocated independently of underlying clinical circumstances. In practice, early milk expression often occurs in the setting of cesarean delivery, maternal–infant separation, ineffective latch, poor milk transfer, prematurity, or anticipated return to work. These factors are themselves associated with breastfeeding outcomes and are difficult to measure fully and control adequately in observational designs. As a result, the observed associations in term populations should be interpreted primarily as signals of association rather than as strong causal estimates.

From a clinical standpoint, the present review does not support discouraging milk expression in all circumstances. Such a conclusion would be neither evidence-based nor clinically appropriate. Rather, the evidence supports a differentiated approach. In healthy term dyads in which direct feeding at the breast is feasible, routine early substitution with expressed-milk feeding should not be assumed to improve breastfeeding outcomes and may be associated with shorter breastfeeding continuation when it becomes the predominant feeding mode. In preterm, separated, or otherwise pump-dependent dyads, early and frequent expression remains a necessary component of lactation management and appears beneficial for early lactation establishment. Clinicians should therefore document the indication for expression and distinguish expression used to preserve lactation from expression used in place of direct breastfeeding in dyads that might otherwise breastfeed effectively at the breast [29,30].

This review has several strengths. It was restricted to primary studies, focused specifically on early postpartum exposure, included both term and preterm evidence while keeping those populations analytically distinct, and used a structured risk-of-bias framework. The main limitation is that the evidence base itself remains heterogeneous and is dominated by observational designs. In addition, several included studies were descriptive or exposure-characterization studies rather than direct tests of breastfeeding duration, which broadens contextual understanding but reduces etiologic specificity. Finally, because the review question spanned first 24–48 h postpartum, postpartum hospital stay, and early postpartum expression more broadly, some unavoidable conceptual breadth remained within the final corpus. That breadth was necessary to capture the real structure of the literature but should be recognized when interpreting the findings.

Future research should prioritize tighter exposure definitions and clinically coherent comparison groups. Studies should distinguish at minimum between any pump use, hand expression, exclusive expressed milk feeding, mixed direct-plus-expressed feeding, and clinically indicated expression in the setting of separation or prematurity. Prospective studies should measure indication for expression, direct breastfeeding frequency, formula supplementation, maternal breastfeeding intention, and objective markers of early feeding difficulty, because these are likely major confounders. Where feasible, analytic strategies should account explicitly for time-varying exposure and reverse causation. Without these design improvements, the field will continue to generate internally valid results within narrow contexts but inconsistent conclusions at the level of overall breastfeeding policy and postpartum counseling.

In summary, the review supports a context-dependent interpretation: direct breastfeeding remains the preferred and most consistently favorable modality when feasible in healthy term dyads, whereas early and frequent expression is clinically necessary and beneficial for lactation establishment in pump-dependent contexts. The remaining uncertainty concerns causal attribution, because indication for expression and underlying feeding difficulty are difficult to separate from the exposure itself.

## 5. Conclusions

This systematic review shows that early postpartum milk expression is not a single uniform exposure. Associations with breastfeeding outcomes differed according to the exposure family, clinical indication, population type, comparator group, and outcome assessed. The evidence should therefore be interpreted within distinct clinical contexts rather than summarized as a single overall effect of milk expression.

For healthy term mother–infant dyads, the most consistent pattern was that direct feeding at the breast was associated with more favorable breastfeeding continuation than exclusive or predominant expressed breast-milk feeding. This finding does not prove that expressed-milk feeding causes earlier cessation, because most contributing studies were observational and at serious risk of confounding by indication. It does, however, support caution against routine early substitution of direct breastfeeding with expressed-milk feeding when direct breastfeeding is feasible and clinically appropriate.

For preterm, separated, or otherwise pump-dependent dyads, the interpretation is different. In these settings, early and frequent expression is often necessary to establish or preserve lactation, and the available evidence supports benefit for early lactation outcomes such as lactogenesis, milk yield, and achievement of full lactation. Evidence for longer-term breastfeeding continuation in these populations remains less certain.

To sum up, the findings support context-specific lactation counselling. Direct breastfeeding should be actively supported in healthy term dyads, while timely and frequent expression should be supported when direct breastfeeding is delayed or not feasible. Future studies should use precise exposure definitions, document the indication for expression, separate term from preterm populations, and incorporate risk-of-bias-sensitive analyses to strengthen causal interpretation.

## Figures and Tables

**Table 3:** Structured evidence integration by exposure family, population context, risk-of-bias implication, and domain-level interpretation.

Exposure Family/Clinical Context	Main Contributing Studies	Comparative Pattern	Risk-of-Bias Implication	Domain-Level Interpretation
Expression method in healthy term dyads	[13,14,15]	Routine early pumping did not consistently improve later breastfeeding outcomes. Hand expression was more favorable than pumping in one trial, while electric pumping improved short-term milk volume in one comparative study.	Randomized studies had some concerns; the comparative clinical study had serious risk. Short-term milk-volume outcomes were not treated as equivalent to breastfeeding duration.	No domain-level support was found for routine early pumping as a general strategy to improve breastfeeding continuation in healthy term dyads.
Timing and intensity in at-risk, separated, or pump-dependent dyads	[21,22,23]	Earlier initiation and/or more frequent expression was associated with reduced delayed lactogenesis, higher milk yield, or successful full lactation.	Evidence was clinically coherent but limited by some concerns or serious risk, especially in observational pump-dependent settings.	Early and frequent expression appears beneficial for lactation establishment when direct breastfeeding is not immediately feasible; evidence for long-term breastfeeding duration remains less certain.
Expressed-milk feeding modality in healthy term dyads	[12,17,18,20,28]	Exclusive or predominant expressed-milk feeding was generally associated with shorter breastfeeding duration or earlier cessation than direct feeding at the breast; mixed feeding showed less consistent associations.	Most evidence was observational and at serious risk of confounding by indication, exposure complexity, and residual feeding difficulties.	Findings support caution against routine substitution of direct breastfeeding with exclusive expressed-milk feeding in otherwise healthy term dyads.
Composite in-hospital feeding patterns	[19,26]	Direct feeding at the breast during the postpartum hospital stay was associated with more favorable breastfeeding continuation than early exposure to expressed breast milk and/or formula.	Comparator groups were clinically heterogeneous; descriptive studies were contextual rather than causal.	Supports skilled assistance for direct-at-breast feeding during the maternity stay, while recognizing that supplementation or expression may reflect clinical need.
Broad any/ever pump use in population-level studies	[10,16,25,27]	Associations were mixed. Some studies linked pump use with longer breastfeeding duration or documented high prevalence of expression.	Broad exposure definitions combine elective, employment-related, and medically indicated pumping and are not comparable with exclusive expressed-milk feeding or routine early pumping.	Any pump use should not be interpreted as equivalent to routine early pumping or exclusive expressed-milk feeding; these studies mainly contextualize access and use patterns.

**Table 4:** Risk-of-bias assessment of included studies using design-specific appraisal tools (RoB 2 for randomized trials and ROBINS-I for non-randomized comparative studies).

Study	Study Design	Tool	Main Concern(s)	Overall Judgment
[15]	Randomized clinical trial	RoB 2	Some concerns regarding deviations from intended intervention and selective reporting	Some concerns
[14]	Randomized trial	RoB 2	Some concerns regarding deviations from intended intervention and selective reporting	Some concerns
[21]	Randomized trial	RoB 2	Some concerns regarding randomization details and selective reporting	Some concerns
	Cohort study	ROBINS-I	Serious risk of confounding by indication	Serious
[10,27] *	Descriptive observational study	Not formally classified	Descriptive exposure-characterization study	Descriptive/contextual
[13]	Comparative clinical study	ROBINS-I	Serious risk of confounding and participant-selection bias	Serious
[26] *	Descriptive observational study	Not formally classified	Descriptive exposure-characterization study	Descriptive/contextual
[12]	Prospective cohort study	ROBINS-I	Serious risk of confounding by indication and exposure heterogeneity	Serious
[19]	Prospective cohort study	ROBINS-I	Serious risk of confounding and non-equivalent comparator groups	Serious
[28]	Longitudinal survey cohort	ROBINS-I	Serious risk of confounding, self-selection, and exposure complexity	Serious
[25] *	Descriptive cohort study	Not formally classified	Descriptive exposure-characterization study	Descriptive/contextual
[18]	Prospective cohort study	ROBINS-I	Serious risk of confounding and exposure misclassification	Serious
[17]	Prospective cohort study	ROBINS-I	Serious risk of confounding and non-random exposure allocation	Serious
[22]	Prospective observational study	ROBINS-I	Serious risk of confounding in pump-dependent preterm context	Serious
[20]	Cohort study	ROBINS-I	Serious risk of confounding and feeding-pattern complexity	Serious
[16]	Population-based observational study	ROBINS-I	Serious risk of residual confounding despite large sample size	Serious
[23]	Cohort study	ROBINS-I	Serious risk of confounding by clinical severity and lactation support context	Serious

* Studies marked with an asterisk were primarily descriptive or exposure-characterization studies and were retained to contextualize the early postpartum exposure environment. Because these studies did not primarily address a comparative exposure–outcome question, they were not used for causal inference in the main synthesis and were not assigned an overall comparative risk-of-bias judgment using RoB 2 or ROBINS-I.

## Data Availability

No new data were created or analyzed in this study. Data sharing is not applicable to this article.
